# Characteristics and Barriers of Emergency Department Patients Overdue for Cancer Screening

**DOI:** 10.5811/westjem.60400

**Published:** 2023-12-20

**Authors:** Sara W. Heinert, Mohammed Ahmed, Kelvin Guzman-Baez, Ananya Penugonda, Sarah Oh, Affan Aamir, Jeanne M. Ferrante

**Affiliations:** * Rutgers Robert Wood Johnson Medical School, Department of Emergency Medicine, New Brunswick, New Jersey; † Rutgers Robert Wood Johnson Medical School, Department of Family Medicine and Community Health, New Brunswick, New Jersey

## Abstract

**Introduction:**

People without reliable access to healthcare are more likely to be diagnosed with late-stage cancer that could have been treated more effectively if diagnosed earlier. Emergency departments (ED) may be a novel place for cancer screening education for underserved patients. In this study we sought to determine patient characteristics and barriers to cancer screening for those patients who presented to a large, academic safety-net ED and were overdue for breast, cervical, and colorectal cancer screening since the coronavirus 2019 (COVID-19) pandemic.

**Methods:**

Adult ED patients eligible for at least one cancer screening based on US Preventive Serivces Task Force guidelines completed a web-based survey. We examined the association of demographic characteristics and having a personal physician with being overdue on screening using chi-square or the Fisher exact test for categorical variables and *t*-tests for continuous variables.

**Results:**

Of 221 participants, 144 were eligible for colorectal, 96 for cervical, and 55 for breast cancer screening. Of eligible patients, 46% (25/55) were overdue for breast cancer screening, 43% (62/144) for colorectal, and 40% (38/96) for cervical cancer screening. There were no significant characteristics associated with breast cancer screening. Being overdue for cervical cancer screening was significantly more likely for patients who were of Asian race (*P* = 0.02), had less than a high school diploma (*P* = 0.01), and were without a routine checkup within the prior five years (*P* = 0.01). Overdue for colorectal cancer screening was associated with patients not having insurance (*P* = 0.04), being in their 40s (*P* = 0.03), being Hispanic (*P* = 0.01), and not having a primary care physician (*P*=0.01). Of 97 patients overdue for at least one screening, the most common barriers were cost (37%), lack of time (37%), and lack of knowledge of screening recommendations (34%). Only 8.3% reported that the COVID-19 pandemic delayed their screening.

**Conclusion:**

The ED may be a novel setting to target patients for cancer screening education. Future work that refers patients to free screening programs and primary care physicians may help improve disparities in cancer screening and cancer mortality rates for underserved populations.

Population Health Research Capsule What do we already know about this issue?

*People without reliable access to healthcare are more likely to be diagnosed with late-stage cancer that could be treated more effectively if diagnosed earlier.*

What was the research question?

*What are the characteristics of and barriers faced by emergency department (ED) patients overdue for cancer screening?*

What was the major finding of the study?

*Patient characteristics were associated with being overdue for cervical and colorectal cancer screening. Cost (37%), lack of time (37%), and lack of knowledge (33%) were barrier.*

How does this improve population health?

*The ED may be a novel setting to target patients for cancer screening education. Our findings can inform future studies to improve cancer screening disparities.*

## INTRODUCTION

The World Health Organization estimates that 30–50% of cancer deaths could be prevented by modifying or avoiding key risk factors and implementing existing, evidence-based prevention strategies.^1^ Early detection of cancer and management of patients who develop cancer can also reduce the cancer burden.^1^ Over time, overall cancer death rates have decreased^2^; however, racial/ethnic and socioeconomic disparities exist. The rate of new cancer is higher for Whites than Blacks, yet cancer deaths are lower for Whites than Blacks.^3^ Hispanic and Black women have higher rates of cervical cancer than other racial/ethnic groups, and Black women and women with less education have the highest rates of death from cervical cancer.^4^ Additionally, people with less education are more likely to die from colorectal cancer (CRC) before the age of 65 than those with more education.^4^

While several factors contribute to cancer disparities, people without reliable access to healthcare are more likely to be diagnosed with late-stage cancer that could have been treated more effectively if diagnosed earlier.^4^ Patients without insurance are significantly less likely to be up to date with mammography and CRC screening than patients with insurance.^5^ Emergency departments (ED) tend to serve in a safety-net capacity for underserved patients.^6^ Hispanic and Black patients are significantly more likely to report higher ED utilization and no usual source of care than White patients.^7^ Visits to the ED for primary care needs are highest for uninsured and low-income patients, suggesting a lack of access to primary care for these patients.^8^ Thus, many ED patients have no interaction with the healthcare system outside the ED, and they can be difficult to reach for cancer screening interventions.

Past studies of cancer screening adherence for eligible ED patients have found that 12–33% of women were overdue or had uncertain adherence with cervical cancer screening^9^ ^–^ ^11^; 12–46% of women were overdue for breast cancer screening^5^ ^,^ ^10^ ^–^ ^12^; and 17–46% were overdue for CRC screening.^5^ ^,^ ^12^ ^,^ ^13^ The percentage of patients overdue for cancer screenings has been significantly higher for those who have no insurance^5^ ^,^ ^9^ ^,^ ^10^ ^,^ ^13^ or a primary care physician,^9^ ^,^ ^13^ and patients with less education,^5^ ^,^ ^9^ ^,^ ^13^ with mixed findings on the role of race and ethnicity,^5^ ^,^ ^9^ ^,^ ^11^ ^–^ ^13^ However, these studies occurred prior to the coronavirus 2019 (COVID-19) pandemic. Results from large national surveys showed that approximately 55% of respondents reported that they or someone in their household delayed or skipped routine medical care during the pandemic,^14^ ^,^ ^15^ suggesting that rates of being overdue for cancer screening may be higher post-pandemic and/or more disparate for some groups of patients. Our objective in this study was to determine the proportion of patients in a large, diverse, academic safety-net ED who were eligible for and overdue on breast, cervical, and CRC screening, as well as to determine their characteristics and the barriers they faced to obtaining screening. We could find no other studies in the literature that explored patient characteristics since the COVID-19 pandemic or determined barriers to cancer screening among ED patients. Additionally, most past work has not included Spanish-speaking patients.

## METHODS

This was a cross-sectional survey study of patients seen from March–September 2022 at the Robert Wood Johnson University Hospital (RWJUH) ED in New Brunswick, New Jersey, a Level I trauma center and safety-net hospital. The ED treats approximately 71,000 adult (21+ years) patients annually and serves a population of approximately 54% women, 39% Black, and 17% Hispanic patients, with 23% having Medicaid and 16% with no insurance.

### Survey Design

Survey questions included demographics, primary care physician and cancer screening questions from the 2020 Behavioral Risk Factor Surveillance System^18^; personal and family history of cancer and cancer information-seeking questions from the Health Information National Trends Survey 2020^19^; cancer screening barriers adapted from Akinlotan et al, 2017^20^; and delay of healthcare due to COVID-19 questions adapted from the National Health Interview Survey 2021.^21^ The assessment consisted of 21 questions that all participants completed, followed by questions specific to each cancer type (2–10 questions per section) that the participant was eligible for screening (based on age and gender). Additionally, 15 questions asked about barriers to cancer screening, including delays due to the COVID-19 pandemic. The survey was available in English and Spanish.

### Survey Administration

Recruitment fliers were posted in the RWJUH ED, which included a quick response (QR) code and link to the survey on REDCap (Research Electronic Data Capture), a secure, web-based software platform designed to support data capture for research studies hosted at our institution.^16^ ^,^ ^17^ Contact information for the study team was also listed on the flier if patients preferred to complete the survey via phone with a member of the study team. Surveys were initially available (March and April 2022) only through passive recruitment (posted fliers in the ED) due to the pandemic; research assistants (RA) actively recruited patients in the ED starting in May 2022. The RAs used convenience sampling to approach all patients during their recruitment shift and inform them about the study, assess eligibility, and direct eligible and interested patients to the survey. Most recruitment shifts were conducted during regular business hours. Several RAs were fluent in Spanish, aiding in communication with Spanish-speaking patients.

**Figure 1.**
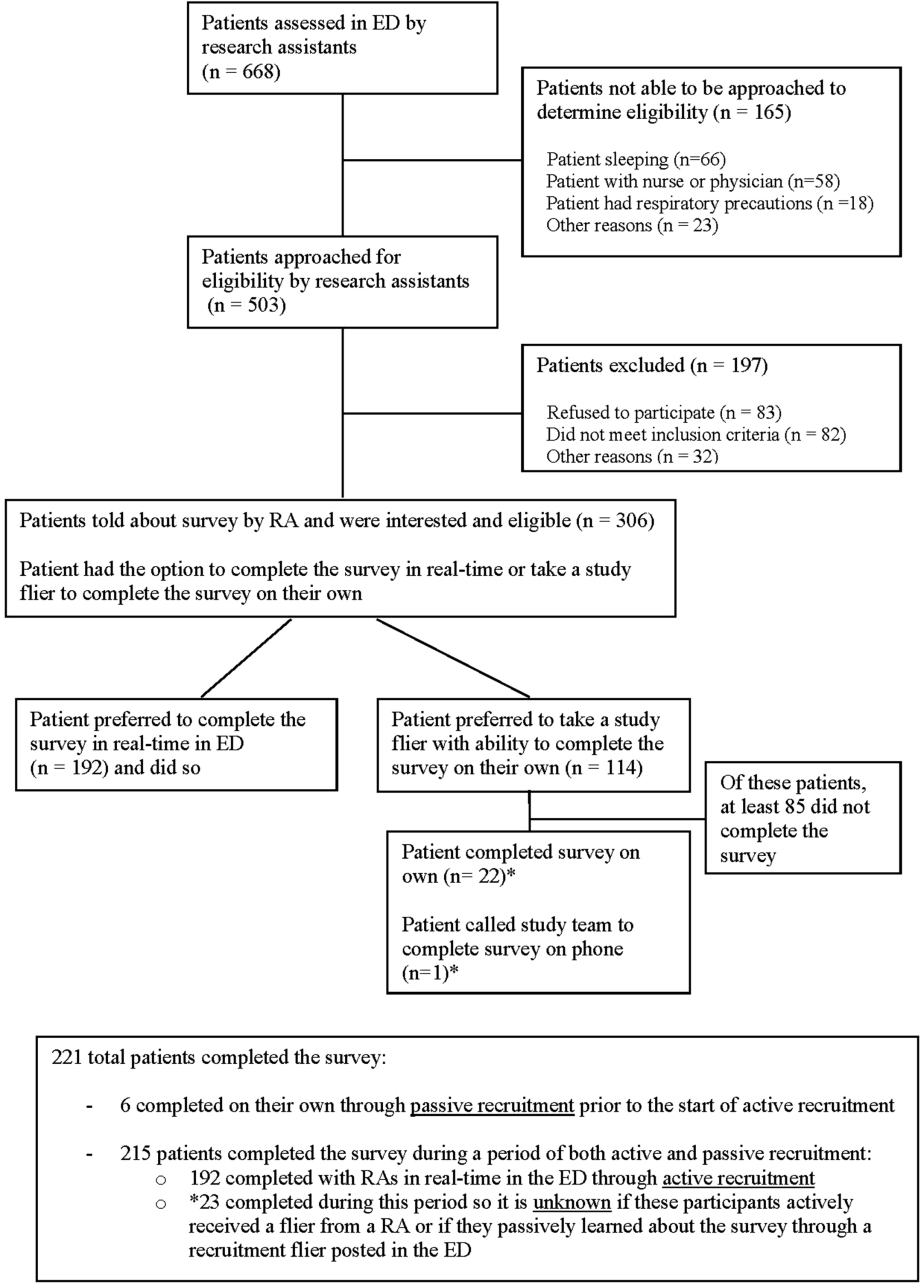
CONSORT diagram for patients approached by a research assistant in the emergency department. RA, research associate; ED, emergency department.

Interested patients had the option to follow the link to the survey on their own devices and complete the survey themselves or, if preferred, have the survey administered to them by the RA. Patients could participate if they were eligible for at least one cancer screening based on US Preventive Services Task Force recommendations for gender and age. ^22^ ^–^ ^24^ Patients self-reported their cancer screening status through the survey. Figure 1 provides details on patients approached by a RA in the ED. Of the three methods to complete the survey, 192 participants completed it when approached by an RA in the ED, 28 completed it on their own via link/QR code from a flier (six during passive recruitment only and 22 during a period of both active and passive recruitment), and one called the study team to complete the survey over the phone. Each participant completed only questions for each cancer type in which they were eligible for screening. All participants received a $15 gift card incentive. The study was approved by the Rutgers University Institutional Review Board.

### Statistical Analysis

For our analytic samples, we excluded any patient who was eligible for screening questions for a cancer type but had previously been diagnosed with that cancer type. For example, if a participant was eligible for breast cancer screening questions, but they had previously been diagnosed with breast cancer then they were excluded from the breast cancer screening data analysis. We excluded women with a hysterectomy from the cervical cancer analysis. We used descriptive statistics to characterize the overall sample and the percentage of participants eligible for each cancer screening, as well as the percentage eligible who were overdue for each cancer screening. We examined bivariate associations of demographic and medical characteristics for participants overdue on screening for each cancer type, using chi-square and the Fisher exact test for categorical variables and *t*-tests for continuous variables. We analyzed data using Stata version 16.0 (StataCorp, College Station, TX). All *P*-values are two-tailed, and α was set at 0.05.

## RESULTS

Characteristics for all 221 survey respondents are found in Table 1. Overall, 14.9% completed the survey in Spanish, 60.2% were women, 18.6% were Black, and 29.9% were Hispanic. The mean age was 51.6 years (SD 15.3), median 55.0. Over 22% and 10% had Medicaid or no insurance, respectively, and about half of participants had a high school education or less (51.2%). About 72% had a primary care physician, and 73% had seen a doctor for a routine checkup within the prior year. A quarter of the sample had a previous history of cancer, and 11% had delayed cancer screening due to the COVID-19 pandemic.

**Table 1.** Survey respondent demographics and health characteristics (N = 221).*

		N	%
Language	English	188	85.1
	Spanish	33	14.9
Gender	Men	88	39.8
	Women	133	60.2
Age (Years)	Mean (SD), median	51.6 (15.3), 55.0	
	18–45	72	32.6
	46–64	103	46.6
	≥65	46	20.8
Race^^^	Black	41	18.6
	Asian	16	7.2
	White	114	51.6
	American Indian/Alaskan Native	2	0.9
	Native Hawaiian or other Pacific Islander	1	0.5
	Other^#^	51	23.1
Ethnicity	Hispanic	66	29.9
	Non-Hispanic	155	70.1
Insurance type	Medicaid	49	22.4
	Medicare	56	25.6
	No insurance	23	10.5
	Private insurance	84	38.4
	Other	7	3.2
Highest level of education	Some high school	32	14.5
	High school degree	81	36.7
	College degree	68	30.8
	Postgraduate degree	25	11.3
	Trade school	15	6.8
Do you have one person you think of as your personal doctor or healthcare provider?	Yes, only one	158	71.5
	More than one	18	8.1
	No	44	19.9
	Not sure	1	0.5
About how long has it been since you last visited a doctor for a routine checkup?	Within the past year (any time less than 12 months ago)	162	73.3
	Within the past 2 years (1 year but less than 2 years ago)	19	8.6
	Within the past 5 years (2 years but less than 5 years ago)	14	6.3
	5 or more years ago	10	4.5
	Not sure	12	5.4
	Never	4	1.8
Have you ever been diagnosed as having cancer?	Yes (Any) Breast Cervical Colorectal	57 7 3 9	25.8 3.2 1.4 4.1
Have any of your first- or second-degree biological relatives ever had cancer? (N = 220)	Yes	126	57.3
	No	85	38.6
	Not sure	9	4.1
Was there any time when you delayed getting a cancer screening because of the coronavirus 2019 pandemic?	Yes	25	11.3
	No	194	87.8
	Not sure	2	0.9

* Percentages may not add to 100 due to missing data.

^ Participants could choose all that apply.

# Other include the following: Hispanic (including Latino/a, Dominican, Mexican, Nicaraguan) (n = 37), more than one race (n = 4), Arabic (n=1), Egyptian (n = 1), and blank (n = 8).

Table 2 shows the percentage of participants eligible for each cancer screening type. Most participants (144) were eligible for CRC screening, followed by 96 for cervical and 55 for breast cancer screening. Of eligible patients, 45.5% were overdue for breast, 39.6% for cervical, and 43.1% for CRC screening.

**Table 2.** Percentage of participants eligible for and overdue on cancer screenings by cancer type

	Eligibility^*^	N of ED patients eligible for screening	N (%) of patients eligible who were overdue on screening
Breast	Women, 50–74 years^+^	55	25 (45.5)
Cervical	Women, 21–65 years	96	38 (39.6)
Colorectal (CRC)	Men and women, 45–75 years	144	62 (43.1)

Notes: *Based on US Preventive Services Task Force (USPSTF) recommendations.

+ Survey was conducted prior to USPSTF changing its recommendation to begin breast cancer screening at age 40.

Table 3 shows demographic and medical characteristics of survey respondents who were overdue on cancer screening compared to the total eligible for cancer screening by cancer type. There were no significant characteristics associated with breast cancer screening. Being overdue for cervical cancer screening was significantly more likely for patients of Asian race (*P* = 0.02), patients who had less than high school diploma (*P* = 0.01), and those without a routine checkup within the prior five years (*P* = 0.01). Overdue for CRC screening was associated with not having insurance (*P* = 0.04), patients in their 40s (*P* = 0.03), being Hispanic (*P* = 0.01), and not having a primary care physician (*P* = 0.01). For the continuous age variable, patients overdue for CRC screening were significantly younger (mean 57.6, SD 8.6) compared to patients not overdue for CRC screening (mean 62.7, SD 7.4, not shown) (*P* = <0.001).

**Table 3.** Demographics and medical characteristics of survey respondents overdue on cancer screening compared to total eligible for cancer screening by type.

		Overdue on breast cancer screening N (%)^1^ 25 (45.5)	*P*-value	Overdue on cervical cancer screening N (%)^2^ 38 (39.6)	*P*-value	Overdue on colorectal cancer screening N (%)^3^ 62 (43.1)	*P*-value
Language	English	25 (49.0)	0.12^e^	31 (36.5)	0.11^e^	51 (40.5)	0.10
	Spanish	0 (0.0)		7 (63.6)		11 (61.1)	
Gender	Men	-	-	-	-	35 (44.9)	0.63
	Women	-		-		27 (40.9)	
Age (years)	Mean (SD), Median (IQR)	60.8 (6.3), 60.0	0.15	44.7 (15.5), 48.0	0.17	57.6 (8.6), 57.0	**<0.001**
	21–39	-	0.45	14 (29.2)	0.06^e^	**-**	**0.03**
	40–49	-		5 (35.7)		13 (68.4)	
	50–65	19 (48.7)		19 (55.9)		36 (42.9)	
	66–75	6 (37.5)		-		13 (31.7)	
Race (Select all that apply)	Black	3 (30.0)	0.60^e^	4 (23.5)	**0.02** ^**e**^	9 (33.3)	0.08^e^
	Asian	0 (0.0)		8 (72.7)		3 (42.9)	
	White	17 (51.5)		13 (30.2)		32 (39.0)	
	Other	5 (45.5)		13 (52.0)		18 (64.3)	
Ethnicity	Hispanic	3 (30.0)	0.32^e^	12 (44.4)	0.54	21 (61.8)	**0.01**
	Non-Hispanic	22 (48.9)		26 (37.7)		41 (37.3)	
Insurance type	Medicaid	7 (70.0)	0.43^e^	12 (44.4)	0.06^e^	10 (40.0)	**0.04** ^**e**^
	Medicare	8 (47.1)		2 (28.6)		19 (35.9)	
	No insurance	1 (33.3)		6 (75.0)		9 (69.2)	
	Private insurance	9 (37.5)		16 (31.4)		20 (40.8)	
	Other	0 (0.0)		2 (100.0)		4 (100.0)	
Highest level of education	Some high school	3 (42.9)	0.49^e^	5 (71.4)	**0.01** ^**e**^	14 (58.3)	0.37^e^
	High school degree	13 (59.1)		15 (44.1)		25 (44.6)	
	College degree	6 (31.6)		16 (47.1)		16 (39.0)	
	Postgraduate degree^+^	1 (50.0)		2 (15.4)		3 (25.0)	
	Trade school	2 (40.0)		0 (0.0)		4 (36.4)	
Have a personal doctor or healthcare provider?	Yes, one or more	23 (45.1)	1.00^e^	27 (35.5)	0.11	48 (38.7)	**0.01**
	No	2 (50.0)		11 (55.0)		14 (70.0)	
About how long has it been since you last visited a doctor for a routine checkup?	Within the past year	16 (38.1)	0.22^e^	26 (36.1)	**0.01** ^**e**^	41 (38.7)	0.30^e^
	Within the past 2 years (1–2 years ago)	2 (66.7)		2 (28.6)		8 (61.5)	
	Within the past 5 years (2–5 years ago)	2 (66.7)		2 (22.2)		4 (44.4)	
	5 or more years ago	3 (75.0)		6 (100.0)		3 (60.0)	
	Not sure	2 (100.0)		1 (100.0)		5 (71.4)	
	Never	0 (0.0)		1 (100.0)		1 (25.0)	
Past cancer diagnosis	Yes	8 (47.1)	0.87	9 (64.3)	0.07^e^	17 (37.8)	0.39
	No	17 (44.7)		29 (35.4)		45 (45.5)	
First- or second-degree biological relatives ever had cancer	Yes	15 (45.5)	0.77^e^	19 (32.2)	0.08^e^	34 (40.0)	0.55^e^
	No	9 (42.9)		18 (50.0)		24 (47.1)	
	Not Sure	1 (100.0)		1 (100.0)		4 (57.1)	

Notes: ^+^Includes master’s or doctorate degrees; e = Fisher exact test, ^1^Of 55 total eligible for breast cancer screening, ^2^Of 96 total eligible for cervical cancer screening. ^3^Of 144 total eligible for colocrectal cancer screening.

*IQR*, interquartile range.

There were 97 unique participants who were overdue on at least one screening for which they were eligible. Table 4 summarizes cancer information-seeking and cancer screening barriers for these patients. Of patients overdue on screening, 35.1% had looked for information about cancer, and 77.3% were completely or very confident that they could get advice or information about cancer if they needed it. Most participants would first go to the internet (44.3%) or a doctor (42.3%) if they had a strong need to get information about cancer. The most common barriers to screening were cost (36.8%), lack of time (36.5%), and lack of knowledge regarding screening recommendations (34.4%). Of participants who were overdue on screening, only 8.3% reported that they delayed getting cancer screening because of the COVID-19 pandemic.

**Table 4.** Cancer information-seeking and barriers for patients overdue on breast, cervical, and/or colorectal cancer screening (N = 97)

	Response	Patients overdue on screening N (%)
Ever looked for information about cancer from any source	Yes	34 (35.1)
	No	63 (65.0)
Overall, how confident are you that you could get advice or information about cancer if you needed it?	Completely or very confident	75 (77.3)
Where you would you go first if you had a strong need to get information about cancer	Internet	43 (44.3)
	Doctor or health care provider	41 (42.3)
	Family	6 (6.2)
	Cancer organization	2 (2.1)
	Library	2 (2.1)
	Other	2 (2.1)
	Friends/Co-worker	1 (1.0)
	Books	0 (0.0)
Screening Barriers (Agree or Strongly Agree)	Lack of time	35 (36.5)
	Cost	35 (36.8)
	Not knowing screening recommendations	33 (34.4)
	Fear of finding cancer	32 (33.0)
	Forgetting to schedule appointment	30 (31.6)
	Anxiety	27 (27.8)
	Other health problems	25 (26.3)
	Transportation	22 (22.9)
	Anticipation of pain	21 (21.9)
	Embarrassment	21 (21.9)
	Language barriers	18 (18.8)
	Opposite sex physician	9 (9.4)
Was there any time when you DELAYED getting cancer screening because of the pandemic?	Yes	8 (8.3)
	No	89 (91.8)
Please share how the COVID-19 pandemic delayed you getting a cancer screening. (n=8)	Didn’t want to leave house High-risk patient Increased fatigue, interest, forgetfulness “It just screwed up everything.” Mammogram got rescheduled Process	3 1 1 1 1 1

*COVID-19*, coronavirus 2019.

## DISCUSSION

We examined cancer screening adherence two years since the start of the COVID-19 pandemic and across three types of cancers: breast; cervical; and colorectal. It was not surprising that the highest percentage of ED patients responding to the survey were eligible for CRC screening since that group comprised both men and women. Approximately 40–45% of eligible patients were overdue on breast, cervical, or CRC screening.

Despite the fact that only 8.3% of participants reported that COVID-19 delayed their cancer screening, our findings found relatively high rates of patients overdue on screening compared to past studies conducted in the ED prior to the pandemic (overdue rates 12–33% for cervical,^9^ ^–^ ^11^ 12–46% for breast,^5^ ^,^ ^10^ ^–^ ^12^ and 17–46% for CRC screening^5^ ^,^ ^12^ ^,^ ^13^). Our higher rates of overdue screenings were probably due not to the pandemic but may have been related to the characteristics of our patient population. Our study had much higher percentages of Asian (7%) and Hispanic (30%) participants than other similar studies, which had 1–3%^5^ ^,^ ^9^ ^,^ ^10^ ^,^ ^13^ and 7–18%,^5^ ^,^ ^10^ ^–^ ^13^ ^,^ ^25^ respectively. Additionally, in our study Spanish-speaking patients represented 15% of all participants.

We could only find one previous study of screening adherence in ED patients that mentioned the availability of Spanish-speaking RAs for their survey, but no report of how many of the patients they surveyed spoke Spanish. ^11^The study found 12% overdue for breast and 33% overdue for cervical cancer screening, and had higher rates of White and privately insured participants than our study.^11^ Future research on culturally relevant cancer screening interventions that target Asian and Hispanic patients in the ED are warranted.

No significant characteristics were found for women overdue on breast cancer screening, suggesting that there may be existing programs that provide more equitable access to mammograms for all women. One prior study across five EDs found that being overdue for both breast and cervical cancer screenings was significantly higher for women with no insurance. ^10^ Our findings found similar results for cervical and CRC but not breast cancer screening. Our results suggest other patients who could be potentially targeted in the ED for cervical cancer screening: Asian women; those with less education; and patients not having a routine checkup within the prior five years. For CRC screening, potential populations to target in the ED include patients who are younger (40s), Hispanic, uninsured, and those without a primary care physician.

In addition to patient characteristics, our study also determined barriers to screening for overdue patients. To our knowledge, no other studies have explored barriers to cancer screening in patients presenting to the ED. Cost, lack of time, and lack of knowledge were the most prevalent screening barriers for patients overdue on cancer screenings. Future work can explore more in-depth explanations of these patient barriers and may be helpful for developing future interventions. For example, our findings suggest the ED may be a novel place to educate and refer patients for cancer screenings.

## LIMITATIONS

This study has several limitations. First, it was conducted in one ED; however, the setting is probably similar to other academic safety-net hospitals in the Northeast US. Second, while recruitment fliers were displayed in the ED with a link for any patient interested in completing the survey, most participants (192/ 221) were recruited in person by a RA during business hours. In our convenience sample, Black patients were underrepresented and patients with previous cancer over-represented; thus, our cancer screening rates may be overestimated. Our hospital is affiliated with the only National Cancer Institute-designated cancer center in New Jersey, which could help explain our large percentage of participants with previous cancer diagnosis. It is possible that given their past history of cancer, they may have been more willing to participate in a cancer-related survey, more likely to get cancer screenings even during the pandemic, and may have had characteristics that are different than the general ED population, such as more connectedness to the healthcare system.

Additionally, our small sample precluded multivariable analyses; thus, our findings may have been confounded by other factors. Finally, we implemented both active and passive (fliers posted in the ED) recruitment, but we collected recruitment information only for participants during active recruitment. We do not know the percentage of total ED patients during our study period who were eligible for or received cancer screening, as chart review was beyond the scope of this study. Neither did we link recruitment method type to individual surveys, as all participants completed the survey through the same REDCap survey link. Thus, we were unable to determine whether participant characteristics differed between recruitment method types.

## CONCLUSION

The ED may be a novel setting to target patients for cancer screening education. Our findings can inform future studies to create interventions that incorporate ways to improve cancer screening knowledge and support to improve disparities in cancer screening among ED patients. Referral to free screening programs and primary care physicians may help improve disparities in cancer screening and cancer mortality rates for underserved populations.
